# Ultrasound phase microscopy for rapid label-free super-resolution vascular imaging

**DOI:** 10.21203/rs.3.rs-9488223/v1

**Published:** 2026-05-11

**Authors:** Zhengchang Kou, Junhang Zhang, Chen Gong, Jie Ji, Nathiya Vaithiyalingam Chandra Sekaran, Zikai Wang, Rita J. Miller, Yaoheng Yang, Daniel Adolfo Llano, Qifa Zhou, Michael L. Oelze

**Affiliations:** 1 Beckman Institute for Advanced Science and Technology, University of Illinois at Urbana-Champaign, IL 61801, USA; 2 Alfred E. Mann Department of Biomedical Engineering, University of Southern California, Los Angeles, CA 90089, USA; 3 F.M. Kirby Neurobiology Center, Boston Children’s Hospital, and Department of Neurology, Harvard Medical School, Boston, MA 02115, USA; 4 Department of Electrical and Computer Engineering, University of Illinois at Urbana-Champaign, IL 61801, USA; 5 Department of Molecular and Integrative Physiology, University of Illinois at Urbana-Champaign, IL 61820, USA; 6 Carle Illinois College of Medicine, University of Illinois at Urbana-Champaign, IL 61801, USA; 7 Roski Eye Institute, Keck School of Medicine, University of Southern California, Los Angeles, CA 90089, USA

## Abstract

Mapping deep microvascular structure and dynamics is essential for understanding organ function, neurovascular coupling and disease progression. Ultrasound localization microscopy (ULM) achieves super-resolution vascular imaging in deep tissue, but its dependence on exogenous contrast agents and prolonged stochastic tracking limits throughput and impedes the capture of transient hemodynamics. Here we introduce ultrasound phase microscopy (UPM), a label-free super-resolution ultrasound approach based on spatiotemporal phase decoding. UPM operates entirely on the receive side by converting motion-induced blood-signal phase shifts into sub-wavelength spatial gating through engineered phase gradients. A theoretical analysis shows that the effective gate width scales inversely with the square root of the signal-to-noise ratio, shifting the operative resolution boundary away from the classical diffraction limit. UPM achieves *in vivo* vascular resolution down to 4.8 μm without contrast agents. We apply UPM to whole-brain vascular mapping in a mouse model of Alzheimer’s disease, functional ultrasound imaging of visually evoked hemodynamics, and handheld renal imaging *in vivo*. UPM thus provides a rapid, contrast-free strategy for super-resolved vascular imaging in deep tissue.

## Introduction

The ability to resolve microvascular architecture and flow dynamics in deep tissue is central to understanding organ function, neurovascular coupling and disease progression^1^. Optical methods can visualize capillary networks with high spatial precision, but their penetration depth is fundamentally limited^2^. Ultrasound, by contrast, offers deep penetration and high temporal resolution, making it well suited for in vivo hemodynamic imaging. However, conventional ultrasound flow imaging remains constrained by acoustic diffraction, such that microvascular structures well below the wavelength scale are blurred into unresolved vascular signals.

Ultrasound localization microscopy (ULM) provided a major advance by overcoming this diffraction barrier through the localization and tracking of individual microbubbles across large numbers of frames^3^. ULM has enabled deep-tissue vascular imaging with spatial resolution approaching the capillary scale. Despite this progress, the method depends on exogenous contrast agents and on the stochastic accumulation of sparse microbubble events over extended acquisitions. These requirements limit throughput and make it difficult to capture non-repetitive or rapidly evolving hemodynamic processes. In functional settings, for example, repeated stimulus cycles are often needed to achieve sufficient vascular sampling^4^.

To avoid contrast injections, recent efforts have explored label-free super-resolution imaging using endogenous red blood cells (RBCs) as flow reporters^5,6^. Yet these approaches remain challenged by the dense, coherent and overlapping nature of blood scattering, which makes the isolation and tracking of individual RBCs physically and computationally difficult. Computational strategies based on deep learning^7^ or compressed sensing^8^ offer partial solutions but typically rely on extensive training data or iterative priors. More fundamentally, standard ultrasound flow estimation derives velocity from temporal phase shifts alone^9^. Because these measurements are formed through diffraction-limited receive beamforming, the phase signatures of closely spaced sub-wavelength vessels are blended within a broad lateral point spread function and cannot be resolved into distinct microvascular sources. Here we introduce ultrasound phase microscopy (UPM), a label-free super-resolution ultrasound strategy based on spatiotemporal phase decoding. Rather than localizing individual scatterers, UPM uses receive-side phase engineering to convert displacement-induced blood-signal phase shifts into sub-wavelength spatial gating, thereby shifting the spatial resolution boundary from acoustic diffraction towards a signal-to-noise ratio (SNR) limited regime. This mechanism enables rapid super-resolved vascular imaging without contrast agents or prolonged stochastic tracking. We show that UPM achieves micrometer-scale vascular resolution *in vivo* with markedly shorter acquisitions than localization-based approaches, supports high-throughput whole-brain imaging in a mouse model of Alzheimer’s disease, resolves visually evoked hemodynamics, and is compatible with handheld renal imaging in vivo.

### UPM shifts the effective resolution limit from diffraction to SNR

**Extended Data Fig. 1 |:**
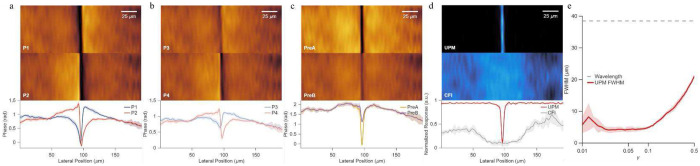
Microbubble-trace validation shows later saturation of UPM resolution gain at higher SNR. **a-c,** Phase responses at successive steps of the UPM process for a selected microbubble trace. **d,** Normalized response comparison for the selected microbubble trace between UPM and CFI. **e,** Measured FWHM of UPM for the selected microbubble trace at different γ values. Resolution improvement saturated at smaller γ values than in the *in vivo* label-free experiment, consistent with the higher SNR of the microbubble-trace data.

**Fig. 1 |:**
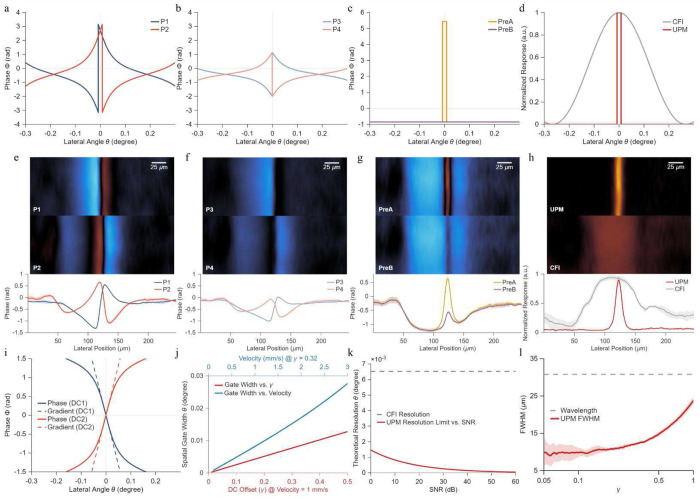
UPM shifts the effective resolution boundary from acoustic diffraction towards SNR. **a-c,** Schematic illustration of the UPM signal formation process. Motion-induced blood-signal phase shifts are modulated by engineered receive-side spatial phase gradients and converted into a sub-wavelength spatial gate through nonlinear phase wrapping and symmetric phase combination. **d,** Comparison of the effective lateral response of UPM and CFI, showing narrowing of the main lobe with UPM. **e-h,** Representative processing steps of UPM for a single microvessel selected from an *in vivo* label-free 50-MHz mouse brain dataset, demonstrating the generation of the phase-derived spatial gate and the resulting narrowing of the vessel response. **i,** Schematic of the paired asymmetric receive apodizations used to generate opposing spatial phase gradients around the focal origin. **j,** Theoretical prediction of the synthesized spatial-gate width on blood velocity and the apodization parameter γ. **k,** Theoretical prediction of the achievable UPM resolution as a function of SNR, showing inverse square-root scaling with SNR **l,** Measured FWHM of a representative microvessel under different γ values, demonstrating saturation of resolution gain at small *γ*.

To overcome the physical constraints of conventional ultrasound flow imaging, UPM achieves label-free super-resolution by shifting the effective resolution boundary from acoustic diffraction to SNR. UPM operates entirely on the receive side, using paired asymmetric apodizations to generate opposing spatial phase gradients around the focal origin (Fig. 1i). When blood flows through the imaging field, relative phase shifts are modulated by these engineered gradients and, through a nonlinear phase-wrapping step followed by symmetric phase combination, converted into an ultra-narrow sub-wavelength spatial gate (Fig. 1a–c). This strategy produces a substantially narrower effective main lobe than conventional color flow imaging (CFI) (Fig. 1d), without localization or tracking of individual scatterers. The width of the synthesized spatial gate depends on both blood velocity and the apodization parameter γ (Fig. 1j) but cannot improve indefinitely as γ decreases. Our theoretical analysis (**Supplementary Discussion 1**) predicts that, rather than following the classical Rayleigh criterion, the achievable resolution scales inversely with the square root of the SNR (Fig. 1k). We validated this framework by resolving a single microvessel from an *in vivo* label-free 50-MHz mouse brain dataset through each stage of the UPM pipeline (Fig. 1e–h), and by measuring vessel full width at half maximum (FWHM) over a range of γ values (Fig. 1l). Additional validation using a microbubble trace experiment with higher SNR (Extended Data Fig. 1) showed that the resolution gain saturated at smaller γ values than in the label-free case, consistent with SNR-limited expectation.

### Label-free micrometer-scale resolution brain vascular imaging with low computational overhead

**Fig. 2 |:**
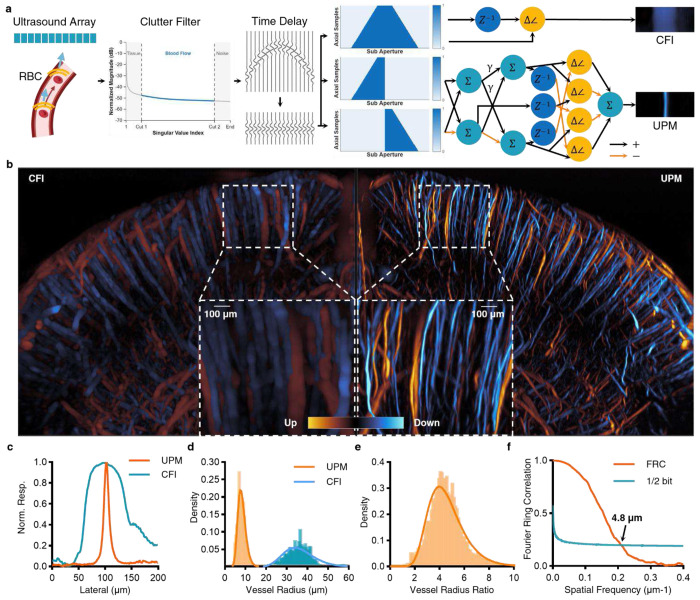
UPM enables label-free micrometer-scale brain vascular imaging with low computational overhead. **a,** Schematic of the computationally efficient UPM beamforming framework. The three apodizations required by UPM are synthesized from two orthogonal base apodizations corresponding to the left and right halves of the receive aperture, enabling DAS-like computational complexity without localization or tracking. **b,** Representative brain vascular maps acquired with UPM and CFI in the same *in vivo* 50-MHz mouse brain dataset, showing improved delineation of dense capillary networks with UPM. **c,** Representative cross-sectional vessel profiles from selected regions in the UPM and CFI images. **d,** Distribution of vessel radius measured from the selected region for UPM and CFI. **e,** Radius ratio analysis showing the reduction in measured vessel radius with UPM relative to CFI. **f,** FRC analysis of the 50-MHz mouse brain dataset. At the 1/2-bit threshold, UPM achieved a global FRC-derived resolution of 4.8 μm.

To translate this framework into practical high-throughput imaging, we implemented UPM using a computationally efficient beamforming architecture for large-field-of-view (FOV) brain mapping (Fig. 2a). By exploiting the linearity of delay-and-sum (DAS) beamforming, the apodizations required by UPM can be synthesized from two orthogonal base apodizations corresponding to the left and right halves of the receive aperture. As a result, UPM retains computational complexity comparable to that of standard DAS (**Supplementary Discussion 2**) while avoiding the iterative localization and tracking steps required by localization-based super-resolution approaches.

**Extended Data Fig. 2 |:**
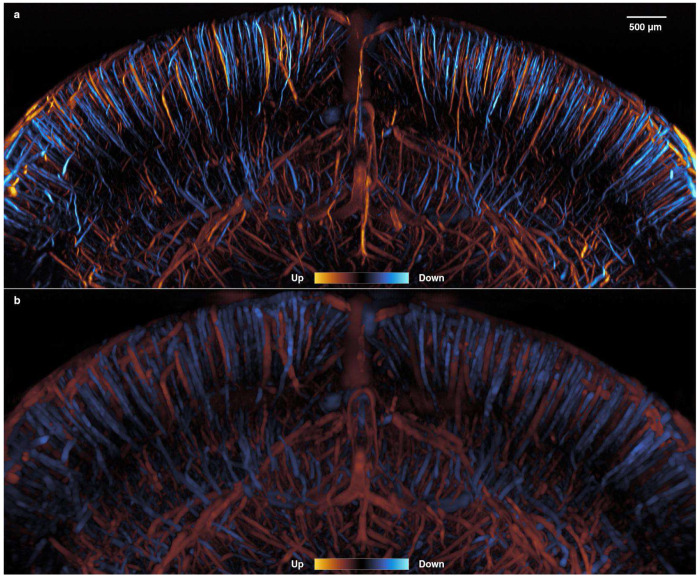
Additional comparison of 50-MHz label-free mouse brain imaging with UPM and CFI. **a,** UPM image from the 50-MHz label-free mouse brain scan. **b,** Corresponding CFI image from the same dataset.

Using a 50-MHz ultrasound transducer array, we performed *in vivo* cerebrovascular mapping of the mouse brain over a volumetric FOV of 9.4 × 32.5 × 4.7 mm^3^, acquiring 65 slices with 4,576 frames per slice in 4.76 s per slice. Direct comparison with conventional CFI showed that UPM resolved dense capillary networks that remained blurred within the diffraction-limited CFI signal (Fig. 2b and Extended Data Fig. 2). Cross-sectional vessel profiles and radius analysis further demonstrated a marked improvement in spatial definition (Fig. 2c, d): UPM measured a mean vessel radius of 8.4 μm, compared with 36.6 μm for CFI, corresponding to a 4.6-fold reduction on average and a maximum radius ratio of 10.5 (Fig. 2e). To quantify global image resolution, we applied Fourier ring correlation (FRC)^10^, a standard metric in super-resolution ultrasound imaging. At the 1/2-bit threshold, FRC yielded a global FRC resolution of 4.8 μm for UPM (Fig. 2f).

**Extended Data Fig. 3 |:**
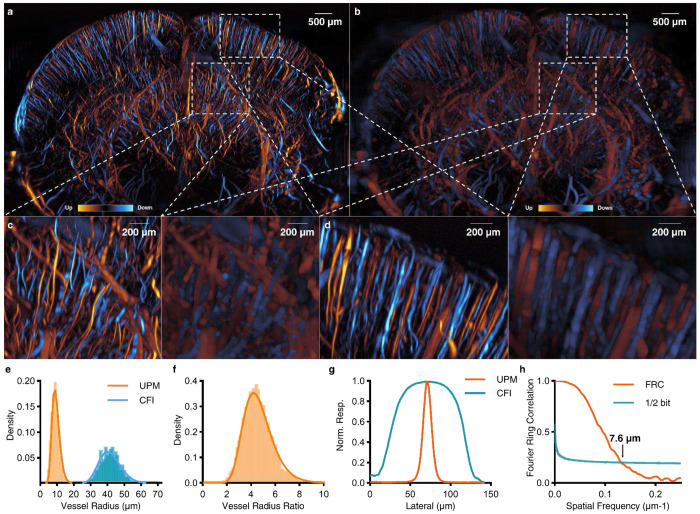
Validation of UPM performance in a label-free mouse brain using a 40-MHz ultrasound array. **a-b,** Representative UPM and CFI images of the 40-MHz label-free mouse brain scan. **c-d,** Enlarged comparisons of the boxed regions from **a, b**, showing improved delineation of fine vessels with UPM. **e-f,** Vessel radius distribution comparison between UPM and CFI, and the corresponding resolution-improvement ratio measured from a selected region of the 40-MHz mouse brain dataset. **g,** Representative single-vessel cross-sectional comparison between UPM and CFI. **h,** FRC analysis of the 40-MHz mouse brain dataset. At the 1/2-bit threshold, UPM achieved a global resolution of 7.6 μm.

**Extended Data Fig. 4 |:**
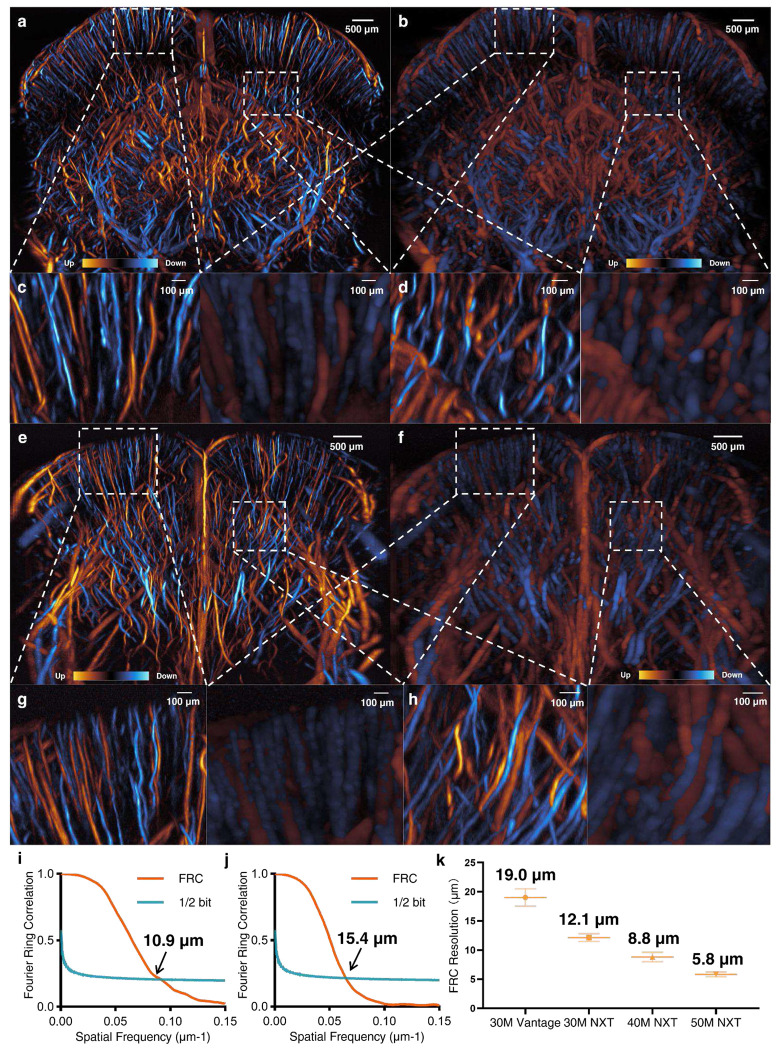
Validation of UPM performance in the mouse brain using 30-MHz ultrasound systems. **a-b,** Representative UPM and CFI images of the 30-MHz label-free mouse brain scan acquired on the new generation ultrasound platform. **c-d,** Enlarged comparisons of the boxed regions from **a, b**. **e-f,** Representative UPM and CFI images of the 30-MHz label-free mouse brain scan acquired on the old generation ultrasound platform. **g-h,** Enlarged comparisons of the boxed regions from **e, f**. **i,** FRC analysis of the 30-MHz mouse brain dataset acquired on the newer platform. At the 1/2-bit threshold, UPM achieved a global resolution of 10.9 μm. **j,** FRC analysis of the 30-MHz mouse brain dataset acquired on the legacy ultrasound platform. At the 1/2-bit threshold, UPM achieved a global resolution of 15.4 μm. **k,** Summary of FRC-derived spatial resolutions measured at the 1/2-bit threshold across all slices in all mouse brain datasets.

We next tested whether this performance generalized across probes and imaging platforms. With a 40-MHz array, UPM mapped mouse brain vasculature over a FOV of 10.2 × 39.5 × 6.3 mm^3^ across 79 slices (Extended Data Fig. 3a–d), again outperforming CFI and reducing the mean vessel radius from 42.2 μm to 9.4 μm, with a maximum radius ratio of 13.5 (Extended Data Fig. 3e, f). FRC analysis yielded a global resolution of 7.6 μm (Extended Data Fig. 3h). With a 30-MHz array, UPM maintained high spatial performance over a FOV of 10.2 × 38.4 × 6.3 mm^3^ across 64 slices, achieving a global resolution of 10.9 μm (Extended Data Fig. 4a–d, i). Finally, on a previous generation ultrasound platform with lower sampling rate and limited buffer capacity, UPM still resolved mouse brain vasculature over a FOV of 6.8 × 72 × 5.5 mm^3^ across 120 slices, with a global resolution of 15.4 μm (Extended Data Fig. 4e–h, j). Across all mouse brain datasets, FRC analysis confirmed consistent performance gains across imaging frequencies and hardware configurations (Extended Data Fig. 3k).

### UPM generalizes across organs, animal models and motion-prone imaging conditions

**Fig. 3 |:**
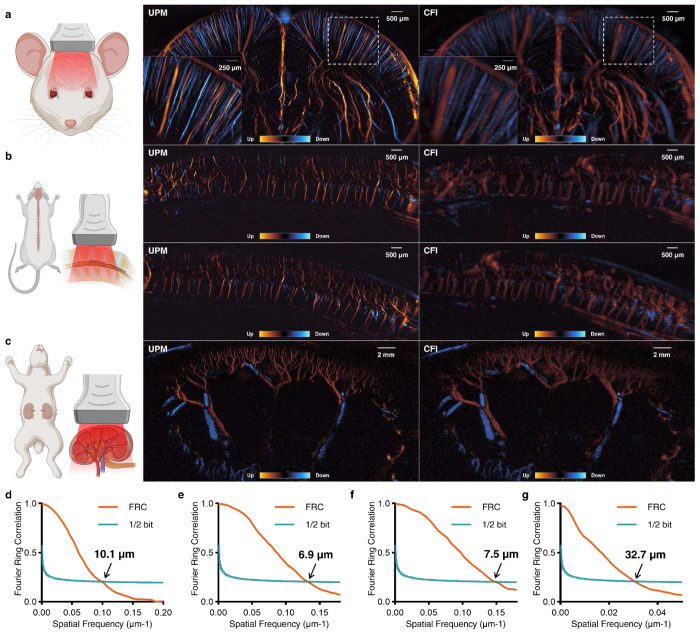
UPM is compatible with multiple organs, animal models and motion-prone imaging conditions. **a,** Representative UPM and CFI images of the rat brain acquired with a 30-MHz array. **b,** Representative UPM and CFI images of the rat spinal cord. **c,** Representative handheld in vivo UPM and CFI images of rabbit kidney acquired under uncompensated physiological motion. **d,** FRC analysis of the rat brain dataset, yielding a spatial resolution of 10.1 μm. **e, f**, FRC analyses of two rat spinal cord segments, yielding spatial resolutions of 6.9 μm and 7.5 μm, respectively. **g,** FRC analysis of the handheld rabbit kidney dataset, yielding a spatial resolution of 32.7 μm despite lower frequency, handheld acquisition and strong physiological motion.

To assess the applicability of UPM across anatomical scales and tissue types, we extended imaging from the mouse brain to larger animal models, including rat brain, rat spinal cord and rabbit kidney. In the rat brain, UPM achieved a FOV of 13.6 × 37.2 × 6.3 mm^3^ across 62 slices using a 30-MHz array and 1,800 frames per slice, with an acquisition time of 2 s per slice. FRC analysis yielded a spatial resolution of 10.1 μm, corresponding to approximately 20% of the acoustic wavelength (Fig. 3a, d). This high spatial fidelity was also maintained in the rat spinal cord, where UPM achieved resolutions of 6.9 μm and 7.5 μm in two different segments (Fig. 3b, e, f), corresponding to approximately sevenfold improvement beyond the wavelength scale.

To evaluate translational feasibility under clinically relevant conditions, we performed in vivo rabbit kidney imaging with a handheld probe. Unlike stage-scanned acquisitions, this setup introduced uncompensated respiratory and cardiac motion and did not use gating or motion correction. Even under these conditions, UPM reconstructed renal microvasculature from an ultrashort 0.16-s acquisition comprising 300 frames, aided by a pulse-inversion harmonic sequence (10-MHz transmit, 20-MHz receive) to improve SNR at depth (Fig. 3c). Over a FOV of 32 × 20.5 mm^2^, UPM achieved a global resolution of 32.7 μm (Fig. 3g), corresponding to more than a twofold improvement beyond the acoustic wavelength scale despite the lower frequency, handheld acquisition and strong physiological motion. These results demonstrate the feasibility of UPM across distinct organs, species and hardware settings, and support its potential for rapid label-free super-resolution vascular imaging in translational settings.

### UPM resolves pathological and functional neurovascular changes in the brain

**Fig. 4 |:**
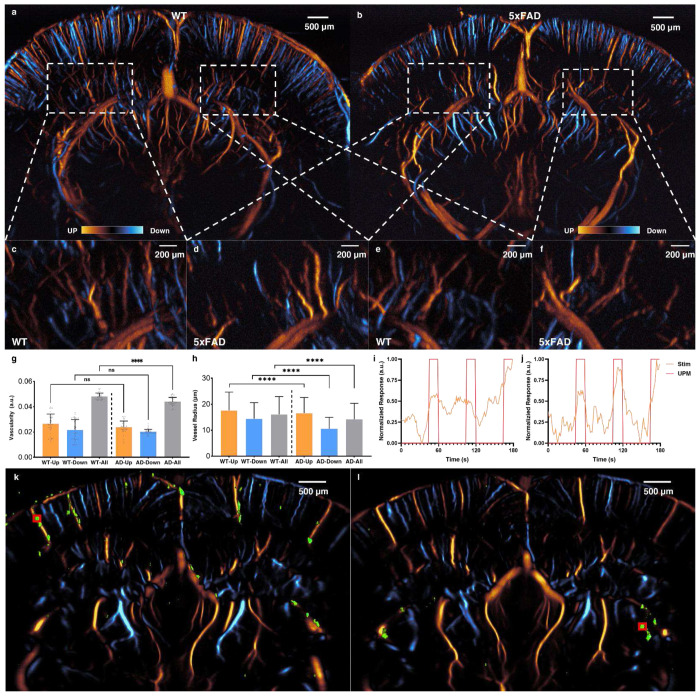
UPM reveals Alzheimer’s disease-associated microvascular changes and microvascular-scale functional hemodynamics in the mouse brain. **a, b,** Representative coronal whole-brain UPM images from a wild-type (WT) mouse (**a**) and a 5xFAD mouse (**b**). Dashed boxes indicate regions shown at higher magnification in **c–f**. **c–f,** Enlarged views of the boxed regions in **a, b,** showing reduced vascularity in 5xFAD relative to WT. **g,** Quantification of hippocampal vascularity for upward-flow, downward-flow and total vasculature in WT and 5xFAD mice. Mean vascularity was lower in 5xFAD mice than in WT mice for both upward and downward flow components, although these individual differences did not reach significance (upward flow, *P* = 0.0599; downward flow, *P* = 0.2692; two-sided Welch’s *t*-test). Total vascularity was significantly lower in 5xFAD mice than in WT controls (two-sided Welch’s *t*-test, *t* = 6.270, d.f. = 76.00, *P* < 0.0001). **h,** Quantification of vessel radius in the hippocampus for upward-flow, downward-flow and combined vessel categories. Vessel radii were significantly smaller in 5xFAD mice than in WT mice in all three categories (all two-sided Welch’s *t*-tests, *P* < 0.0001). **i, j,** Representative regional hemodynamic time courses extracted from the red boxed regions in **k** and **l,** respectively, overlaid with the visual stimulation paradigm**. k, l,** Representative activation maps from two coronal planes during visual stimulation fUS imaging, overlaid on the corresponding UPM vascular maps. Green pixels indicate stimulus-correlated activation. Correlation coefficients between the regional UPM time series and the stimulation paradigm were 0.5394 and 0.8076 for the rostro lateral visual area (k) and lateral geniculate nucleus (l), respectively, across 120 paired time points for each region.

Beyond structural imaging, UPM resolved both disease-associated microvascular changes and stimulus-evoked hemodynamics in the mouse brain. To assess its sensitivity to cerebrovascular pathology, we imaged whole brains from wild-type (WT) and 5xFAD mice using a 30-MHz ultrasound array. Across a volumetric field of view of 10.2 × 24.6 × 6.3 mm^3^, 41 slices were acquired, with 1,792 frames collected over 2 s at each slice. Compared with WT mice, UPM revealed reduced hippocampal vascularity and altered vessel radius in the 5xFAD brain (Fig. 4a–f). Alterations that were difficult to resolve with diffraction-limited imaging became quantifiable with UPM, whose micrometer-scale resolution enabled measurement of both vascularity (Fig. 4g) and vessel radius distributions (Fig. 4h).

To quantify these changes, we manually segmented the hippocampal region across all slices. Mean vascularity in the hippocampus was lower in 5xFAD mice than in WT mice for both upward and downward flow components, although these individual differences did not reach significance (upward flow, *P* = 0.0599; downward flow, *P* = 0.2692; two-sided Welch’s *t*-test; Fig. 4g). In contrast, overall hippocampal vascularity was significantly lower in 5xFAD mice than in WT controls (two-sided Welch’s *t*-test, *t* = 6.270, d.f. = 76.00, *P* < 0.0001; Fig. 4g). Vessel radius analysis further revealed significantly smaller vessel radius in 5xFAD mice across the upward-flow, downward-flow and combined vessel categories (all two-sided Welch’s *t*-tests, *P* < 0.0001; Fig. 4h), consistent with remodeling of the hippocampal microvasculature in AD.

We next examined whether UPM could capture functional hyperemia with comparable spatial specificity. During visual stimulation fUS imaging, ultrafast ultrasound data were acquired repeatedly every 1.5 s, with each acquisition consisting of 640 frames collected over 0.8 s and yielding one UPM image. Each trial comprised 45 s of baseline followed by 15 s of stimulation, and three trials were performed in each experiment. Two datasets were acquired from coronal planes separated by 0.2 mm. Correlation of the UPM time series with the stimulation paradigm revealed localized activation in the rostrolateral visual area and lateral geniculate nucleus, with correlation coefficients of 0.5394 and 0.8076, respectively, across 120 paired time points for each region (Fig. 4k, l). The corresponding regional hemodynamic traces are shown in Fig. 4i, j. Whereas conventional fUS typically generates activation maps that cannot distinguish vessels, UPM localized activation-induced hyperemia to discrete microvascular branches, enabling separation of hemodynamic signals from individual vessels within deep brain regions.

## Discussion

Here we present UPM, a label-free super-resolution microvessel imaging method that combines short acquisition, low computational burden and microvascular-scale resolution over a large field of view. By decoding flow information through spatial phase gating, UPM enables super-resolution microvascular imaging without the localization or tracking of individual microbubbles or erythrocytes. In doing so, it addresses three major constraints that have limited the broader use of super-resolution ultrasound imaging: dependence on exogenous contrast agents, long acquisition times and high computational costs.

A key advance of UPM is that it achieves super-resolution under label-free conditions while substantially reducing the acquisition burden relative to localization-based approaches. Using the same probe and imaging platform as in a previous study^11^, UPM achieved global vascular resolution down to 7.6 μm from seconds of acquisition in the 40-MHz mouse brain dataset, whereas prior contrast-enhanced super-resolution imaging required minutes of data collection to reach a comparable scale of resolution. Although the physical basis of UPM differs fundamentally from localization-based ultrasound microscopy, these results indicate that phase-encoded flow information can provide an efficient and complementary route to super-resolved vascular mapping. Validation on additional probes and imaging platforms further supports the applicability of the method across different experimental configurations.

The translational feasibility of UPM is illustrated by the handheld, label-free imaging experiments in rabbit kidney, in which a large FOV was obtained with subwavelength vascular resolution from an ultrashort acquisition. This acquisition regime markedly reduces data size and reconstruction demands, and is compatible with imaging scenarios in which motion, workflow simplicity, and throughput are critical. Although the current beamforming time remains longer than acquisition (**Supplementary Table 3**), the computational structure of UPM is favorable for acceleration because it does not require iterative localization or particle tracking. Continued advances in GPU- and FPGA-based beamforming^12^, together with increased parallelization of reconstruction pipelines, should further reduce latency and may support near-real-time implementation.

More broadly, UPM expands the scope of super-resolution ultrasound from structural angiography towards integrated vascular phenotyping. In the present study, UPM resolved both disease-associated microvascular changes in the 5xFAD brain and visually evoked hemodynamic responses during functional ultrasound imaging. These results suggest that UPM can bridge mesoscale hemodynamic imaging and microvascular readouts, enabling quantitative assessment of vascular remodeling, neurovascular coupling and flow-direction-resolved responses within the same framework. Such capability could be valuable in neuroscience, where longitudinal monitoring of cortical and subcortical microcirculation is increasingly important, as well as in renal imaging and transplantation, where rapid label-free assessment of microvascular perfusion may inform tissue viability and graft function. More generally, UPM may provide a practical platform for functional ultrasound, biomarker development and preclinical therapeutic evaluation.

Several limitations should be considered. First, because UPM relies on relative phase offsets induced by the displacement of RBCs and benefits from high-frequency ultrasound to maximize RBCs’ backscatter, its performance remains coupled with SNR, penetration depth and hardware sensitivity. This may limit applications in deep organs, large subjects or poorly perfused tissues. Second, although UPM is more tolerant to short acquisitions than many existing super-resolution approaches, the method still depends on sufficient phase stability and may be affected by substantial physiological or probe motion, particularly in non-gated or freehand acquisitions. Third, the achievable resolution depends not only on frequency and acquisition duration but also on flow rate and frame rate, and its behavior should be characterized more systematically across vascular geometries, flow regimes and organ systems. Fourth, while the functional results demonstrate sensitivity to localized hemodynamic changes, UPM measures vascular responses rather than neuronal activity directly and therefore remains subject to the interpretational constraints inherent to hemodynamic imaging. Finally, broader clinical translation will require prospective evaluation across anatomical sites, disease settings and patient populations to establish reproducibility, robustness and diagnostic utility.

Taken together, these findings establish UPM as a distinct strategy for super-resolution ultrasound imaging that is label-free, rapid and computationally efficient. By relaxing the requirements for contrast administration, prolonged acquisition and intensive post-processing, UPM may broaden access to super-resolved vascular imaging in both preclinical research and translational settings.

## Methods

### Animal Preparation

#### Mouse brain (50-MHz and 30-MHz):

A 2-month-old C57BL/6J mouse was anesthetized with 5% isoflurane for induction and maintained under 2% isoflurane via a nose cone. Anesthesia depth was monitored via pedal reflex and respiratory rate. To prevent corneal dehydration, GenTeal^®^ Tears Lubricant Eye Gel (Alcon, Fort Worth, TX, USA) was applied to both eyes. The animal was positioned on a temperature-controlled heating pad throughout the procedure. The craniotomy was performed with a lateral width of 6 mm and an anterior-posterior extent from bregma to lambda. The exposed brain surface was covered with ultrasound gel to ensure acoustic coupling. All experimental procedures were approved by the Institutional Animal Care and Use Committee at the University of Southern California.

#### Mouse brain (40-MHz):

Mouse anesthesia was induced using ketamine/xylazine anesthetics, and then mice were placed in a stereotaxic frame with a nose cone supplying oxygen for maintenance. Lidocaine (1%) was intradermally injected into the scalp to supplement anesthesia. Ear bars were used to secure the mouse head to the stereotaxic imaging stage. The scalp of the mouse was removed, and a cranial window was opened on the left and right sides of the skull using a rotary Dremel tool, starting at the sagittal suture and moving laterally to expose the lateral expanse of the cerebral cortex. The animal use protocol was approved by the Institutional Animal Care and Use Committee at the University of Illinois at Urbana-Champaign.

#### Rat brain and spine:

A 6-month-old Long-Evans rat was anesthetized with 3% isoflurane for induction and maintained under 2% isoflurane via a nose cone. Anesthesia depth was monitored via pedal reflex and respiratory rate. To prevent corneal dehydration, GenTeal^®^ Tears Lubricant Eye Gel (Alcon, Fort Worth, TX, USA) was applied to both eyes. For brain imaging, the craniotomy extended from 4 mm anterior to bregma through the bregma-to-lambda interval along the anterior–posterior axis, with a lateral width of 12 mm. The exposed brain surface was covered with ultrasound gel to ensure acoustic coupling. For spine imaging, a midline incision was made after shaving the dorsal skin, and the paraspinal muscles were removed. A laminectomy spanning vertebral levels L1–L4 was performed to expose the spinal cord. Ultrasound gel was applied, and the transducer was positioned along the longitudinal axis of the spinal cord (in the direction of the spinal nerves). All experimental procedures were approved by the Institutional Animal Care and Use Committee at the University of Southern California.

#### Rabbit kidney:

One in vivo trial was performed with a 3.5-month-old female New Zealand White rabbit weighing 5.8 kg (Charles River Laboratories, Wilmington, MA). The animal use protocol was approved by the Institutional Animal Care and Use Committee at the University of Illinois at Urbana-Champaign. Anesthesia was induced with 5% isoflurane and maintained with 2% isoflurane, both via face mask. The level of anesthesia was monitored by pedal reflex and respiratory rate. Ophthalmic ointment was applied bilaterally, and the rabbit was placed on a heating pad to maintain body temperature. The skin over the left kidney was shaved. The left kidney was scanned transabdominally by handheld VisualSonics MS200 probe.

#### AD/WT mouse:

6-month-old male 5xFAD and wild-type (WT) C57BL/6J mice were anesthetized with 5% isoflurane for induction and maintained under 2% isoflurane via a nose cone. Anesthesia depth was monitored via pedal reflex and respiratory rate. To prevent corneal dehydration, GenTeal^®^ Tears Lubricant Eye Gel (Alcon, Fort Worth, TX, USA) was applied to both eyes. The animals were positioned on a temperature-controlled heating pad throughout the procedure. The craniotomy was performed with a lateral width of 6 mm and an anterior-posterior extent from bregma to lambda. The exposed brain surface was covered with ultrasound gel to ensure acoustic coupling. All experimental procedures were approved by the Institutional Animal Care and Use Committee at the University of Southern California.

#### Mouse brain functional ultrasound:

Prior to the experiment, the animals were dark-adapted for 12 hours. To record light stimulation activities in the visual system, a full-field strobe flash using a Grass Photic stimulator (Grass Instrument Co., W. Warwick, RI, USA) was placed 10 cm away from the eye to deliver visual stimulation. A function generator (AFG 3252C, Tektronix, Beaverton, OR, USA) was utilized to generate a single cycle burst to synchronize the Verasonics system and the flash stimulator. Specifically, the trigger-out port of the Verasonics system was connected to the trigger-in port of the function generator, and the output port of the function generator was connected to the trigger-in port of the Grass Photic stimulator. During the stimulation phase, the Verasonics system generated a trigger signal every 1.5 s, which prompted the function generator to output a 6-Hz square wave, driving the strobe to flash at a frequency of 6 Hz. The visual stimulation paradigm consisted of three repetitive cycles of 45-s rest and 15-s stimulation.

### Ultrafast Ultrasound Data Acquisition

Ultrafast ultrasound data were acquired using commercialized ultrasound imaging research systems and ultrasound linear arrays without modification in hardware.

#### NXT System:

##### 50-MHz mouse brain vascular imaging:

A Visual Sonics MS700 linear array that has 256 elements and a center frequency of 50-MHz was used. All elements (total width = 9.4 mm) of the probe were used. An ultrafast plane-wave imaging sequence (19 tilted plane waves, −13.5° to 13.5° in 1.5° step size, pulse-repetition frequency PRF=18,240 Hz) was developed to acquire ultrafast ultrasound data at a compounded frame rate of 960 Hz. A 50-MHz, 1.5 cycle pulse was generated by the ultrasound system as the transmit signal. Each acquisition has 768 16-bit quantization samples in fast time with a sampling frequency of 125-MHz. The ultrasound data were transferred to a host computer (Dell Precision 7960 with Intel Xeon W7-3565X and 512 GB DDR5 RAM) via PCI-Express Gen3 x16 interface and stored to a NVME SSD array (RAID 0 with 4x Samsung 990 Pro 4TB). 4,576 compounded frames (86,944 acquisitions) were acquired for each imaging plane (4.76 seconds acquisition for each imaging plane).

The imaging array was fixed on a motorized stage to translate in elevational direction with a step size of 50.8 μm.

##### 40-MHz mouse brain vascular imaging:

A Visual Sonics MS550S linear array that has 256 elements and a center frequency of 40-MHz was used. The center 192 elements (total width = 10.2 mm) of the probe were used. An ultrafast plane-wave imaging sequence (15 tilted plane waves, −14° to 14° in 2° step size, pulse-repetition frequency PRF=15,360 Hz) was developed to acquire ultrafast ultrasound data at a compounded frame rate of 1024 Hz. A 41.67-MHz, 1.5 cycle pulse was generated by the ultrasound system as the transmit signal. Each acquisition has 1024 16-bit quantization samples in fast time with a sampling frequency of 125-MHz. The ultrasound data were transferred to a host computer (Dell Precision 5860 with Intel Xeon W7-2595X and 256 GB DDR5 RAM) via PCI-Express Gen3 x16 interface and stored to a NVME SSD array (RAID 0 with 8x Samsung 990 Pro 2TB). 4,096 compounded frames (61,440 acquisitions) were acquired for each imaging plane (4 seconds acquisition for each imaging plane). The imaging array was fixed on a motorized stage to translate in elevational direction with a step size of 50 μm.

##### 30-MHz mouse brain vascular imaging:

A Visual Sonics MS550D linear array that has 256 elements and a center frequency of 30-MHz. The center 192 elements (total width = 10.2 mm) of the probe were used. An ultrafast plane-wave imaging sequence (15 tilted plane waves, −14° to 14° in 2° step size, pulse-repetition frequency PRF=15,360 Hz) was developed to acquire ultrafast ultrasound data at a compounded frame rate of 1024 Hz. A 31.25-MHz, 1.5 cycle pulse was generated by the ultrasound system as the transmit signal. Each acquisition has 1024 16-bit quantization samples in fast time with a sampling frequency of 125-MHz. The ultrasound data were transferred to a host computer (Dell Precision 7960 with Intel Xeon W7-3565X and 512 GB DDR5 RAM) via PCI-Express Gen3 x16 interface and stored to a NVME SSD array (RAID 0 with 4x Samsung 990 Pro 4TB). 4,096 compounded frames (61,440 acquisitions) were acquired for each imaging plane (4 seconds acquisition for each imaging plane). The imaging array was fixed on a motorized stage to translate in elevational direction with a step size of 50.8 μm.

##### Microbubble trace experiment:

A small number of microbubbles (Lantheus DEFINITY) were used in the microbubble trace experiment, where the microbubbles were pushed away from the transducer face and recorded over time. The same imaging setup used for the 40-MHz mouse brain scan was used. Instead of acquiring 4,096 frames, only 512 frames were acquired in the microbubble trace experiment. A fixed transmit voltage at 6V was used to avoid microbubble collapsing.

#### Vantage System:

##### 30-MHz mouse brain vascular imaging:

A Visual Sonics MS550D linear array that has 256 elements and a center frequency of 30-MHz. The center 128 elements (total width = 6.8 mm) of the probe were used. An ultrafast plane-wave imaging sequence (9 tilted plane waves, −18° to 18° in 4.5° step size, PRF=14,400 Hz) was developed to acquire ultrafast ultrasound data at a compounded frame rate of 800 Hz. A 31.25-MHz, 1.5 cycle pulse was generated by the ultrasound system as the transmit signal. Each acquisition has 1280 14-bit quantization samples in fast time with a sampling frequency of 125-MHz (interleave sampling mode was used). The ultrasound data were transferred to a host computer (Dell Precision 5860 with Intel Xeon W7-2595X and 512 GB DDR5 RAM) via PCI-Express Gen3 x16 interface and stored to a NVME SSD array (RAID 0 with 4x Samsung 990 Pro 4TB). 1,920 compounded frames (17,280 acquisitions) were acquired for each imaging plane (2.4 seconds acquisition for each imaging plane). The imaging array was fixed on a motorized stage to translate in elevational direction with a step size of 60 μm.

##### Rat brain and spinal cord vascular imaging:

A Visual Sonics MS550D linear array that has 256 elements and a center frequency of 30-MHz. All 256 elements (total width = 13.6 mm) of the probe were used. An ultrafast plane-wave imaging sequence (9 tilted plane waves, −4° to 4° in 1° step size, PRF=16,200 Hz) was developed to acquire ultrafast ultrasound data at a compounded frame rate of 900 Hz. A 31.25-MHz, 1 cycle pulse was generated by the ultrasound system as the transmit signal. Each acquisition has 1024 (768 for spine imaging) 14-bit quantization samples in fast time with a sampling frequency of 125 MHz (interleave sampling mode was used). The ultrasound data were transferred to a host computer (HP Z6A G5 with AMD Threadripper Pro 7985WX and 256 GB DDR5 RAM) via PCI-Express Gen3 x16 interface and stored to a NVME SSD (Samsung 9100 Pro 4TB). 1,800 compounded frames (16,200 acquisitions) were acquired for each imaging plane (2 seconds acquisition for each imaging plane). The imaging array was fixed on a motorized stage to translate in elevational direction with a step size of 60 μm.

##### Rabbit kidney vascular imaging:

A Visual Sonics MS200 linear array that has 256 elements and a center frequency of 15-MHz. All elements of the probe (total width = 32 mm) were used. An ultrafast plane-wave imaging sequence (9 tilted plane waves, −4° to 4° in 1° step size, PRF=33,333 Hz) was developed to acquire ultrafast ultrasound data at a compounded frame rate of 1852 Hz. A pair of 10.83-MHz full cycle pulses with opposite polarity were generated by the ultrasound system for each steering angle and the received signals from both pulses were accumulated inside the Verasonics system to implement pulse inversion and reduce the data size. A 10.83-MHz, 1 cycle pulse was generated by the ultrasound system as the transmit signal. Each acquisition has 1664 14-bit quantization samples in fast time with a sampling frequency of 62.5 MH. The ultrasound data were transferred to a host computer (Dell Precision 5820 with Intel Xeon W-2255 and 128 GB DDR4 RAM) via PCI-Express Gen3 x16 interface and stored to a NVME SSD (WD AN1500 1TB). 300 compounded frames (5,400 acquisitions) were acquired for each imaging plane (162 milliseconds acquisition).

##### AD/WT mouse brain vascular imaging:

A Visual Sonics MS550D linear array that has 256 elements and a center frequency of 30-MHz. The center 192 elements (total width = 10.2 mm) of the probe were used. An ultrafast plane-wave imaging sequence (9 tilted plane waves, −18° to 18° in 4.5° step size, PRF=16,128 Hz) was developed to acquire ultrafast ultrasound data at a compounded frame rate of 896 Hz. A 31.25-MHz, 1.5 cycle pulse was generated by the ultrasound system as the transmit signal. Each acquisition has 1024 14-bit quantization samples in fast time with a sampling frequency of 125 MHz (interleave sampling mode was used). The ultrasound data were transferred to a host computer (Dell Precision 5860 with Intel Xeon W7-2595X and 512 GB DDR5 RAM) via PCI-Express Gen3 x16 interface and stored to a NVME SSD array (RAID 0 with 4x Samsung 990 Pro 4TB). 1,792 compounded frames (16,128 acquisitions) were acquired for each imaging plane (2 seconds acquisition for each imaging plane). The imaging array was fixed on a motorized stage to translate in elevational direction with a step size of 60 μm.

##### Mouse brain functional imaging:

A Visual Sonics MS550D linear array that has 256 elements and a center frequency of 30-MHz. The center 128 elements (total width = 6.8 mm) of the probe were used. An ultrafast plane-wave imaging sequence (9 tilted plane waves, −18° to 18° in 4.5° step size, PRF=14,400 Hz) was developed to acquire ultrafast ultrasound data at a compounded frame rate of 800 Hz. A 31.25-MHz, 1.5 cycle pulse was generated by the ultrasound system as the transmit signal. Each acquisition has 1024 14-bit quantization samples in fast time with a sampling frequency of 125-MHz (interleave sampling mode was used). The ultrasound data were transferred to a host computer (Dell Precision 5860 with Intel Xeon W7-2595X and 512 GB DDR5 RAM) via PCI-Express Gen3 x16 interface and stored to a NVME SSD array (RAID 0 with 4x Samsung 990 Pro 4TB). 640 compounded frames (5,760 acquisitions) were acquired for each power Doppler frame. The time interval between two power Doppler frames was 1.5 seconds.

### Ultrasound Phase Microscopy Process

After the acquisition of ultrafast ultrasound data, the reconstruction of label-free super-resolution microvessel image was performed using the UPM algorithm. The UPM process is composed of three steps, which are clutter filtering, beamforming, and phase subtraction.

First the raw channel data are filtered by singular value decomposition (SVD) filter to remove static tissue signal.^13^ Then the filtered ultrasound channel data are delayed and summed with two sets of apodizations: *Apod_left_* and *Apod_right_*. For *Apod_left_*, all the weights on the left half of the sub aperture are 1 and all the weights on the right half of the sub aperture are 0. For *Apod_right_*, all the weights on the right half of the sub aperture are 1 and all the weights on the left half of the sub aperture are 0.

Two samples are beamformed at each pixel location following the process below:

(1) $$\left[\begin{array}{c} S_{\mathrm{left}}\\ S_{\mathrm{right}} \end{array}\right]={\left[\begin{array}{ll} {\mathrm{Apod}}_{\mathrm{left}} & {\mathrm{Apod}}_{\mathrm{right}} \end{array}\right]}^{T}\left[\begin{array}{ll} R_{\mathrm{delayed}} & R_{\mathrm{delayed}} \end{array}\right]$$

where *S_left_* and *S_right_* are the two beamformed samples, and*R_delayed_* is a column vector that contains delayed data samples across the sub aperture. A fixed f-number of 1 was used to determine the sub aperture size for beamforming samples at different depths. The beamformed samples at the same pixel location across different tilted angles are summed together for coherent compounding. The same process was repeated for each pixel location and each frame to finish the ultrafast ultrasound beamforming. Then the three sets of beamformed samples (*S_zm_*, *S*_*dc*1_, and *S*_*dc*2_) needed in the relative phase offset calculation can be synthesized from *S_left_* and *S_right_* which can be found in **Supplementary Discussion 2**.

The relative phase offset calculation is performed following the process below:

(2) $$P_{1}=\sum_{n=1}^{nf-1}\text{arg}\left({\overset{ˇ}{S}}_{dc1}^{n+1}*{\left(-{\overset{ˇ}{S}}_{dc2}^{n}\right)}^{*}\right)$$

(3) $$P_{2}=\sum_{n=1}^{nf-1}\text{arg}\left({\overset{ˇ}{S}}_{dc2}^{n+1}*{\left(-{\overset{ˇ}{S}}_{dc1}^{n}\right)}^{*}\right)$$

(4) $$P_{3}=\sum_{n=1}^{nf-1}\text{arg}\left({\overset{ˇ}{S}}_{dc1}^{n+1}*{\left({\overset{ˇ}{S}}_{zm}^{n}\right)}^{*}\right)$$

(5) $$P_{4}=\sum_{n=1}^{nf-1}\text{arg}\left({\overset{ˇ}{S}}_{dc2}^{n+1}*{\left(-{\overset{ˇ}{S}}_{zm}^{n}\right)}^{*}\right)$$

(6) $$P_{\mathrm{PreA}}=\left(P_{1}+P_{2}\right)$$

(7) $$P_{\mathrm{PreB}}=\left(P_{3}+P_{4}\right)$$

(8) $$P_{\mathrm{UPM}}=P_{\mathrm{PreA}}-P_{\mathrm{PreB}}$$

Where *nf* is the number of frames within one data set and *n* is the frame index.

*P_PreA_* and *P_PreB_* are the two precursors of *P_UPM_*. The final images are generated by averaging *P_UPM_* through all the frames acquired on the same imaging plane.

The above process was performed on an HPE ML350 Gen12 server (Dual Intel Xeon 6517P 16-core processor, 256 GB DDR5 RAM and Nvidia RTX Pro 6000 Blackwell GPU). Customized UPM beamforming software was built with CUDA and compiled as Matlab MEX functions. Fixed DC offset at 0.32 was selected empirically for the weights used in the mouse brain, rat brain, and rat spine imaging. For rabbit kidney imaging, a fixed DC offset at 0.72 was used.

Final images are displayed with adaptive dynamic range according to the standard deviations of the pixels^13^.

### Quantitative Measurement

Skeletonization was performed using Matlab function *bwskel* and the microvessel radius was measured by finding the minimum Euclidean distance between a voxel on the skeletonized vessel to the voxel that has half value of itself.

### Statistical Analysis

Statistical analyses were performed on the measurement units indicated for each dataset. For hippocampal vascularity, comparisons between WT and 5xFAD groups were performed across segmented slices (n = 41 slices per group) using two-sided unpaired Welch’s t-tests. Upward-flow vascularity was compared between WT and 5xFAD slices (t = 1.914, d.f. = 68.08, P = 0.0599; mean difference = −0.002711, 95% CI = −0.005538 to 0.0001159; η^2^ = 0.05104), downward-flow vascularity was compared similarly (t = 1.119, d.f. = 44.54, P = 0.2692; mean difference = −0.001469, 95% CI = −0.004115 to 0.001176; η^2^ = 0.02733), and total vascularity was compared using the same test (t = 6.270, d.f. = 76.00, P < 0.0001; mean difference = 0.004180, 95% CI = 0.002853 to 0.005508; η^2^ = 0.3410). For vessel-radius analysis, comparisons between WT and 5xFAD groups were performed at the vessel level using two-sided unpaired Welch’s t-tests. Upward-flow vessels were compared using n = 9,688 WT vessels and n = 9,374 5xFAD vessels (t = 11.22, d.f. = 18,780, P < 0.0001; mean difference = −1.060, 95% CI = −1.245 to −0.8750; η^2^ = 0.006655), downward-flow vessels using n = 8,820 WT vessels and n = 6,004 5xFAD vessels (t = 44.55, d.f. = 14,799, P < 0.0001; mean difference = −3.869, 95% CI = −4.040 to −3.699; η^2^ = 0.1183), and all vessels combined using n = 18,508 vessels per group (t = 27.46, d.f. = 36,550, P < 0.0001; mean difference = 1.848, 95% CI = 1.716 to 1.980; η^2^ = 0.02022). For functional ultrasound analyses, stimulus-locked responses were evaluated for each ROI using two-sided paired t-tests across n = 120 paired time points. In the rostrolateral visual area, the paired comparison yielded t = 5.43, d.f. = 119, P < 0.0001, with a mean paired difference of −0.1831 (95% CI = −0.2497 to −0.1165; partial η^2^ = 0.1993). In the lateral geniculate nucleus, the paired comparison yielded t = 4.33, d.f. = 119, P < 0.0001, with a mean paired difference of −0.1114 (95% CI = −0.1624 to −0.06044; partial η^2^ = 0.1360). Pearson correlation coefficients between the UPM time series and the stimulation paradigm were r = 0.5394 and r = 0.8076 for the rostrolateral visual area and lateral geniculate nucleus, respectively. All tests were two-sided unless otherwise stated.

## Extended Data

## Supplementary Material

This is a list of supplementary files associated with this preprint. Click to download.

- Supplement.docx
- 50MMouse.mp4
- 40MMouse.mp4
- 30MMouse.mp4
- 30MRatBrain.mp4
- 30MRatSC1.mp4
- 30MRatSC2.mp4

## Data Availability

The acquisition and processing code is available upon request.

## Data availability:

Due to the data size limit, the raw ultrasound data and results data are available upon request.
